# Theoretical investigation of fast illicit drug detection via ternary photonic crystals

**DOI:** 10.1038/s41598-026-39408-4

**Published:** 2026-04-02

**Authors:** B. A. Mohamed, Arafa H. Aly, M. Mobarak, A. S. Shalaby, Walied Sabra

**Affiliations:** 1 TH-PPM group, Physics Department, Beni-Suef University, Beni Suef, Egypt; 2 Physics Department, Beni-Suef University, Beni Suef, Egypt

**Keywords:** Quantum dots, Photonic band gaps, Illicit drugs, Sensitivity, Transfer matrix method, Refractive-Index, Transmittance, Biosensor, AlₓGa₁₋ₓN where x denotes the mole fraction of aluminum, Chemistry, Materials science, Nanoscience and technology, Optics and photonics, Physics

## Abstract

This study presents a theoretical investigation of a highly sensitive optical sensor based on a one-dimensional ternary photonic crystal (1D-TPC) for rapid detection of illicit drugs. The proposed structure consists of alternating layers of polyaniline (PANI), lead sulfide (PbS), and AlₓGa₁₋ₓN quantum dots, incorporating a central defect layer that serves as the sensing region. The detection mechanism relies on monitoring shifts in the defect-mode wavelength induced by variations in the refractive index of different illicit substances. Numerical simulations were carried out using the transfer matrix method (TMM) to analyze the transmission characteristics of the structure under various operating conditions. The influence of key parameters—including aluminum mole fraction, defect layer thickness, incident angle, and number of periods—was systematically investigated. The results demonstrate pronounced tunability of the defect mode and a strong dependence of sensor performance on structural optimization. A maximum sensitivity of 5219.29 nm/RIU was achieved under optimal conditions, indicating excellent detection capability. The obtained results confirm that the proposed photonic crystal sensor offers high sensitivity, strong spectral response, and reliable performance, making it a promising platform for label-free and rapid detection of illicit drugs in forensic and biomedical applications.

## Introduction

The widespread misuse of controlled substances has become a major global challenge, posing serious threats to public health, societal stability, and law enforcement systems^1^. Over recent years, both drug consumption and production have increased substantially, with approximately 269 million people reported to have used drugs in 2018—representing nearly a 30% rise compared to 2009. In parallel, global cocaine production reached an unprecedented level of 1,723 metric tons in 2020^2^. According to reports from the United Nations Office on Drugs and Crime , this growth has been accompanied by a notable increase in drug seizures, particularly involving cocaine, methamphetamine, MDMA, and opioids^2^. Such trends have significantly intensified the workload placed on border control and law enforcement agencies, highlighting the growing complexity of drug monitoring efforts. As a result, there is an increasing demand for rapid, accurate, and cost-effective detection technologies capable of supporting effective drug control and prevention strategies.

Conventional detection methods such as gas chromatography, mass spectrometry, high-performance liquid chromatography, electrochemiluminescence, surface ionization, and surface-enhanced Raman spectroscopy are widely used due to their high analytical accuracy. However, these techniques often suffer from several limitations, including long analysis times, high operational costs, and reliance on bulky and complex laboratory equipment, which restrict their suitability for rapid and on-site detection^3^. To overcome these limitations, optical sensing technologies based on photonic crystals (PhCs) have gained increasing attention owing to their capability for real-time monitoring, compact integration, and cost-effective operation^4^. Photonic crystals are periodic optical structures characterized by the presence of photonic band gaps (PBGs), which are wavelength regions where light propagation is prohibited due to Bragg scattering and interference effects within the periodic lattice^5,6^. This intrinsic ability to manipulate and confine light at the nanoscale makes PhCs highly attractive for sensing applications^7^. Among various photonic crystal configurations, one-dimensional photonic crystals (1D-PCs) are particularly advantageous due to their simple fabrication process, ease of theoretical modeling, low production cost, and broad applicability in photonic devices, making them more practical than higher-dimensional structures^8^. Moreover, semiconductor quantum dots are nanoscale crystalline materials that exhibit unique photochemical and photophysical properties not found in bulk materials or isolated molecules. Their tunable optical behavior, strong light–matter interaction, and excellent photostability make them highly suitable for advanced sensing and optoelectronic applications^9^.

These nanoscale, quantum-confined materials exhibit high luminescence efficiency, strong resistance to photodegradation, and the capability to generate multicolor emissions under a single excitation source^9^. In recent years, quantum dots have gained significant attention as key elements in the fabrication of advanced sensing platforms. Their wide absorption bandwidths coupled with sharp, symmetric emission peaks make high-quality quantum dots especially suitable for optical encoding, multiplexed detection, and diverse optoelectronic applications. Moreover, the incorporation of quantum dots into photonic architectures has been widely explored, resulting in improved device performance and, in some cases, enabling entirely new optical functionalities^9–12^.

This study proposes the design of a one-dimensional ternary photonic crystal structure composed of PANI, PbS, and Al_x_Ga_1-x_N quantum dots for the detection of Illicit drugs by analyzing shifts in defect mode and transmittance intensity. The sensor’s performance is assessed by varying the defect layer thickness, incident angle, mole fraction, and the number of periodic layers. The results underscore the potential of this structure in developing high-performance drug detection systems.

### Formation of the structure and theoretical method

**Fig. 1:**
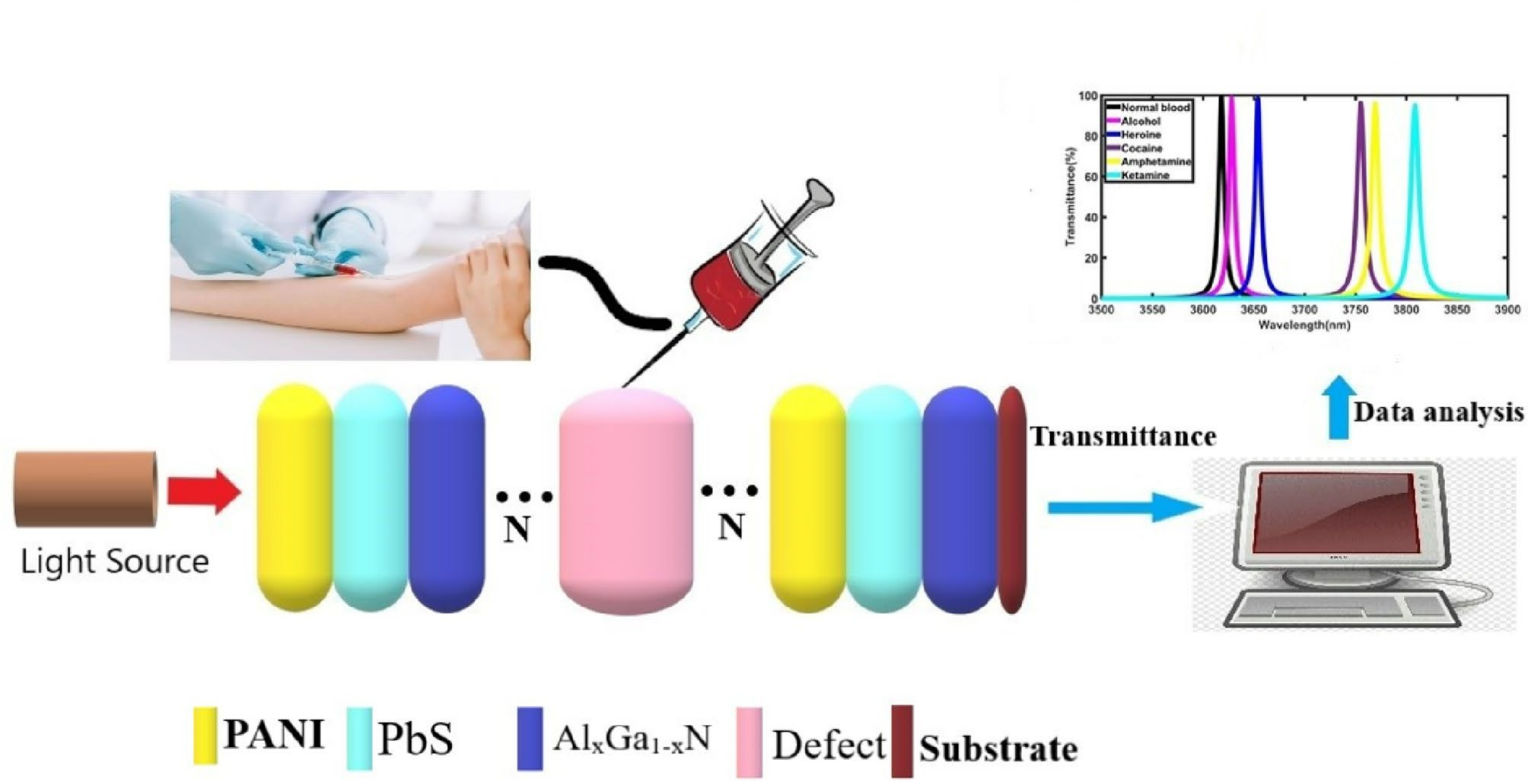
Structural design of proposed 1D-TPC with single defect layer.

The proposed photonic biosensor is made up of 1D-TPC, as shown in Fig. 1. The 1D-TPC is composed of periodic layers of three distinct materials: PANI, PbS, and Al_x_Ga_1-x_N quantum dots, where x denotes the mole fraction of aluminum. These materials deposited one over the other by repeating PANI, PbS, and Al_x_Ga_1-x_N layers N times. A central defect layer is incorporated within the 1D-TPC structure, initially filled with normal human blood (standard sample) characterized by a refractive index of 1.350. This defect medium is then sequentially replaced with individual samples of illicit substances including alcohol, heroin, cocaine, amphetamine, and ketamine in order to evaluate the sensor’s ability to detect and distinguish between these drugs based on their optical responses. The entire structure is encapsulated by air (refractive index n₀ = 1) as the incident medium and glass as the substrate. The final configuration of the 1D-TPC structure is arranged as: air / (PANI / PbS / Al_x_Ga_1-x_N)^N^ / Defect / (PANI / PbS / Al_x_Ga_1-x_N)^N^ / glass, where N represents the number of periods. The refractive indices of the three constituent layers PANI, PbS, and Al_x_Ga_1-x_N are denoted as n_1_, n_2_, and n_3_, with corresponding values of n_1_=1.31, n_2_=4.19, and n_3_= (5.2-1.5x)^0.5^ respectively, where x denotes the mole fraction of aluminum^12,13^. In addition, the thicknesses of the three layers and the defect layer are represented by d_1_, d_2_, d_3_, and dd, with respective values of 100 nm, 155 nm, 450 nm, and 800 nm. The structure is modeled using TMM, which is effective in analyzing multilayer optical systems. The overall structure can be represented by the matrix given below^14^:

1 $${\mathrm{M}}_{Structure} = \left( {\begin{array}{*{20}c} {m_{11} } & {m_{12} } \\ {m_{21} } & {m_{22} } \\ \end{array} } \right) = \left( {m_{A} \,m_{B} \,m_{C} } \right)^{N} m_{D} \left( {m_{A} \,m_{B} \,m_{C} } \right)^{N}$$

Where ${m}_{A}{,m}_{B}$ ${,m}_{C}$ and ${m}_{D}$ denote the characteristic matrices corresponding to the three constituent layers of 1D-TPC and the defect layer, respectively. The elements ${m}_{11},{m}_{12},{m}_{21}, {m}_{22}$ represent the parameters of the overall transfer matrix. The characteristic matrix of a single layer ${m}_{i}$, ($i$ = 1,2, 3, and D layers) is defined as follows^15^:

2 $$m_{i} = \,\left[ {\begin{array}{*{20}c} {\cos (z_{i} )} & {jf_{i}^{ - 1} \sin (z_{i} )} \\ {jf_{i} \sin (z_{i} )} & {\cos (z_{i} )} \\ \end{array} } \right]$$

3 $${z}_{i}=\frac{2\pi {d}_{i}}{\lambda } n_{i}\mathit{cos}({\theta }_{i})$$

In this context, ${f}_{i}={n}_{i} cos({\theta }_{i})$ corresponds to TE polarization, while ${f}_{i}={n}_{i}/ cos({\theta }_{i})$ applies to TM polarization^16^. The parameters ${d}_{i}$​ and ${n}_{i}$​ represent the thickness and refractive index of the i^th^ layer, respectively, while ${\theta }_{i}$ denotes the angle of refraction, determined according to Snell’s law. Ultimately, the transmission coefficient (T) is derived from the matrix elements presented in Eq. (4), as follows^16^:

4 $$\mathrm{T}=\frac{2{f}_{o}}{{f}_{o}{m}_{11}+{f}_{o}{f}_{t}{m}_{12}+{m}_{21}+{{f}_{t}m}_{22}}$$

Equations (5) and (6) characterize ${f}_{o}$ and ${f}_{t}$ for the transverse electric wave (TE) representing the primary medium (air) and final medium (glass) respectively within the proposed structure^17^.

5 $${f}_{o}={ n}_{o}\mathit{cos}({\theta }_{o})$$

6 $${f}_{t}={ n}_{t}\mathit{cos}({\theta }_{t})$$

Accordingly, the transmittance ($T{\prime}$) can be expressed as follows^17^:

7 $$T^{\prime}=\frac{{f}_{t}}{{f}_{o}} {\left|\mathrm{T}\right|}^{2}*100$$

In the subsequent discussion, a comprehensive analysis of the fundamental performance parameters that critically influence the operational efficiency of the proposed sensor will be presented. These parameters include sensitivity (S), quality factor (Q-factor), figure of merit (FOM), signal-to-noise ratio (SNR), dynamic range (DR), limit of detection (LOD), and sensor resolution (SR). Sensitivity represents a key performance indicator for any biosensor, characterizing its ability to detect changes in the surrounding medium. It is quantitatively defined as the amount of wavelength shift per unit change in the refractive index^18^.

8 $${\mathrm{S}}\,{ = }\,\frac{\Delta \lambda }{{\Delta n}}$$

Where, the $\Delta \lambda$ and $\Delta n$ represent the change in resonant wavelength and refractive index, respectively. Another essential performance parameter is the Q-factor, which serves as an indicator of the sharpness of the resonance peak. A higher Q-factor signifies a narrower spectral bandwidth, typically resulting from efficient light confinement within the defect layer. Mathematically, the Q-factor is defined as the ratio of the resonance wavelength (λ) to the full width at half maximum (FWHM) of the corresponding peak, and is expressed as follows^18^:

9 $$\text{Quality factor }=\frac{\uplambda }{\mathrm{FWHM}}$$

The FOM serves as a critical indicator of the sensor’s precision in detecting refractive index variations. It is calculated as the ratio of the sensor’s sensitivity to the FWHM of the resonance peak, as illustrated in the following equation^18^:

10 $$\mathrm{FOM}=\frac{\mathrm{S}}{\mathrm{FWHM}}$$

The SNR measures the clarity of the sensor’s response by comparing the resonance wavelength change (Δλ) to the FWHM. A higher SNR indicates more reliable and accurate detection. It is known mathematically as follows^19^:

11 $${\mathrm{SNR}}\,{ = }\,\frac{\Delta \lambda }{{{\mathrm{FWHM}}}}$$

The DR of a sensor is calculated by dividing the central wavelength of the defect mode by the square root of its FWHM as follows^20^:

12 $$\mathrm{DR}=\frac{\uplambda }{\sqrt{\mathrm{FWHM}}}$$

The LOD defines the minimum detectable change in the sample’s refractive index. The LOD value for our structure is calculated using the following equation^20^:

13 $$\mathrm{LOD}=\frac{\uplambda }{20*\mathrm{S}*\mathrm{Q}-\mathrm{factor}}$$

Finally, the SR represents the minimal spectral dip shift that can be precisely measured by the sensor, and is defined as follows^21^:

14 $$\mathrm{SR}=\frac{2\text{ FWHM}}{3{(\mathrm{SNR})}^\frac{1}{4}}$$

## Results and discussion

This section presents a detailed evaluation of the sensing performance of the proposed device for the detection of illicit drug substances. In addition, the influence of key structural and optical parameters on the system response is systematically examined. These parameters include the aluminum composition ratio in the quantum dot material, the angle of incidence, the thickness of the defect layer, and the number of periodic layers within the structure. Their combined impact on the optical behavior of the sensor is analyzed to optimize detection efficiency and sensitivity.

### Photonic band gap and defect mode analysis

**Fig. 2:**
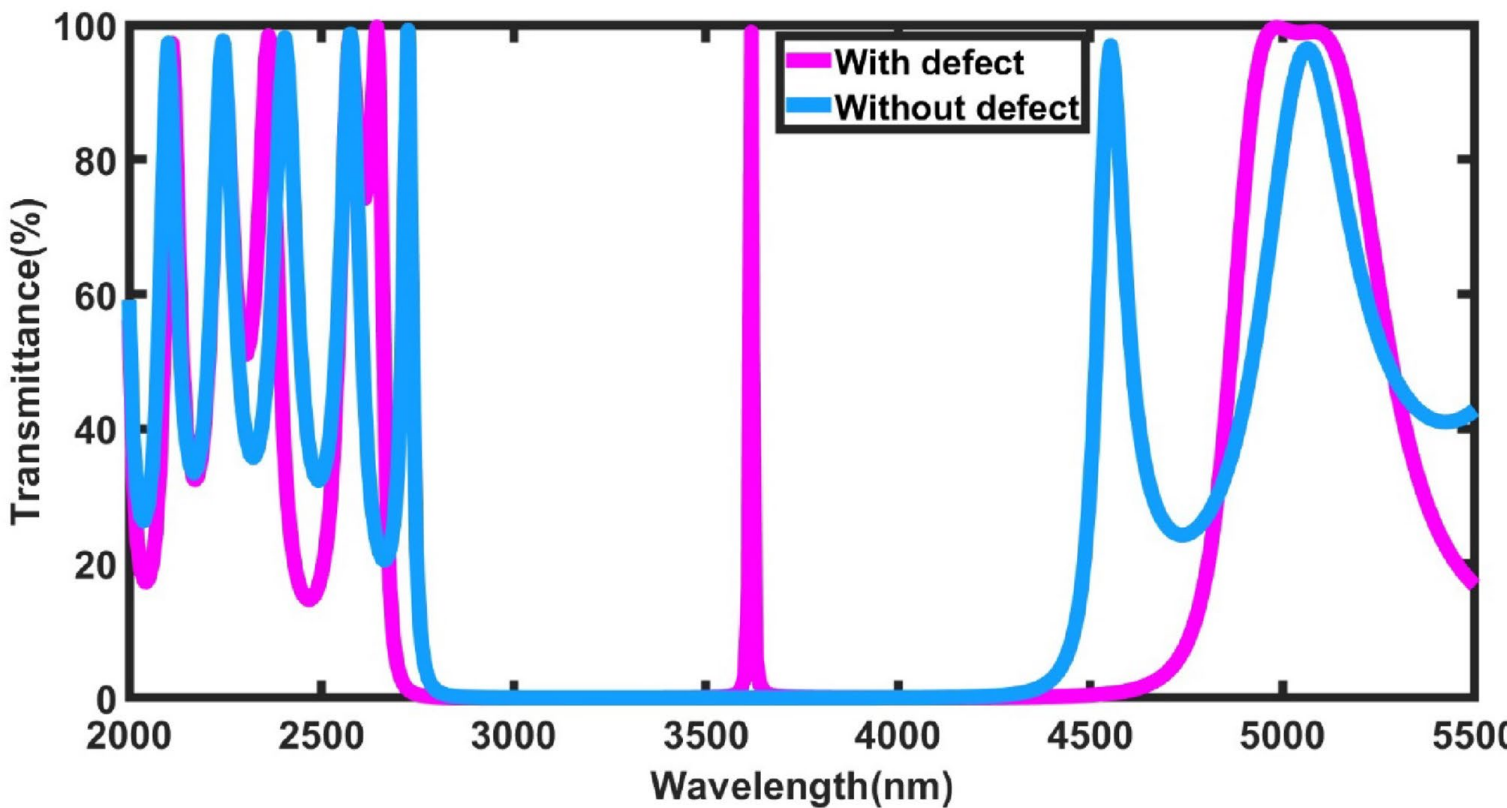
The transmittance spectra of the proposed 1D-TPC without / with defect layer.

In this section, the optical transmission characteristics of the proposed one-dimensional ternary photonic crystal (1D-TPC) sensor designed for illicit drug detection are analyzed. Under normal light incidence, with a defect layer thickness of 800 nm, a molar fraction of x = 0.9, and a periodicity number of N = 4, Fig. 2 depicts the transmittance spectrum as a function of wavelength. The figure compares two structural configurations of the photonic crystal: the first without a defect layer (blue curve), and the second incorporating a defect layer filled with a normal human blood sample (magenta curve), highlighting the effect of the defect on the spectral response of the sensor.

The transmission spectrum exhibits a well-defined photonic band gap (PBG), where the transmittance drops to nearly zero over the wavelength range from 2727.52 nm to 4552.67 nm. This behavior originates from strong destructive interference within the periodic structure, leading to almost complete reflection of incident light in this spectral region. After introducing the blood-filled defect layer, the optical response changes noticeably, and a sharp transmittance peak appears inside the PBG at 3618.01 nm. This peak represents a localized defect mode that does not exist in the defect-free configuration. The presence of this mode indicates that the defect layer enables resonant light transmission at a previously forbidden wavelength. Such resonance arises from light confinement within the defect region, resulting in a pronounced transmission enhancement reaching approximately 98.88% .

### Sensitivity to drug detection

**Fig. 3:**
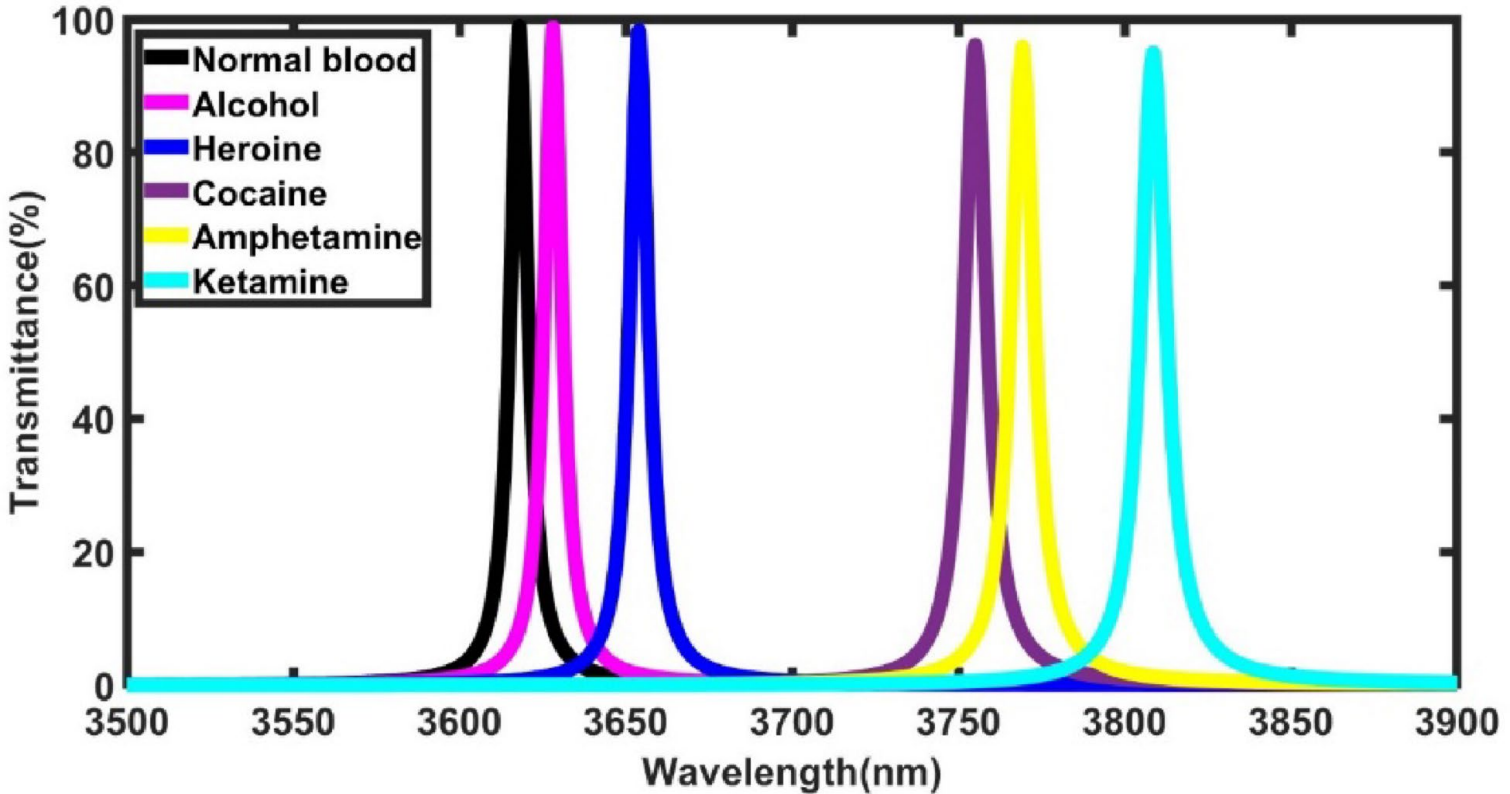
Transmittance spectra showing six defect modes corresponding to normal blood and illicit drugs samples, where x=0.9 mol, N=4, and Incident angle = 0°.

To assess the sensor’s ability to detect various illicit substances, the defect layer medium was sequentially replaced with individual drug samples, including alcohol, heroin, cocaine, amphetamine, and ketamine. The refractive indices corresponding to each substance are presented in Table 1. The results presented in Fig. 3 demonstrate a distinct shift in the position of the defect peak corresponding to each drug sample, indicating the sensor’s ability to effectively differentiate between the substances. This shift indicates that the defect peak position moves to longer wavelengths as the refractive index of the defect layer increases. This behavior can be attributed to the increased optical path length caused by higher refractive index values, resulting in resonance conditions at longer wavelengths as well illustrated in Eqn. 3. Table 2 shows the numerical values ​​of the observed shifts in the defect peak position and sensitivity corresponding to each sample under investigation at normal incidence of light, defect layer thickness of 800 nm and x=0.9 mol, N=4. Notably, the sensitivity values, which range between 890.35 and 901.05 nm/RIU, demonstrate that the sensor exhibits a high level of responsiveness to changes in the defect layer’s refractive index, making it suitable for detecting even slight variations caused by different drug samples.

**Table 1:** Refractive indices values of various illicit drugs under investigation.

**Drug name**	**Refractive index**	**Ref**
Alcohol	1.3614	^22^
Heroine	1.3901	^22^
Cocaine	1.5022	^23^
Amphetamine	1.518	^23^
Ketamine	1.562	^23^

**Table 2:** Numerical values of the defect mode position and corresponding sensitivity for a normal blood and samples of illicit drugs under investigation at x= 0.9 mol.

**Sample**	**Defect mode position (nm)**	**Sensitivity (nm/ RIU)**
Normal blood	3618.01	-
Alcohol	3628.16	890.35
Heroine	3653.99	897.25
Cocaine	3755.15	901.05
Amphetamine	3769.36	900.89
Ketamine	3808.63	899.15

### Effect of Al composition (Mole Fraction x)

**Fig. 4:**
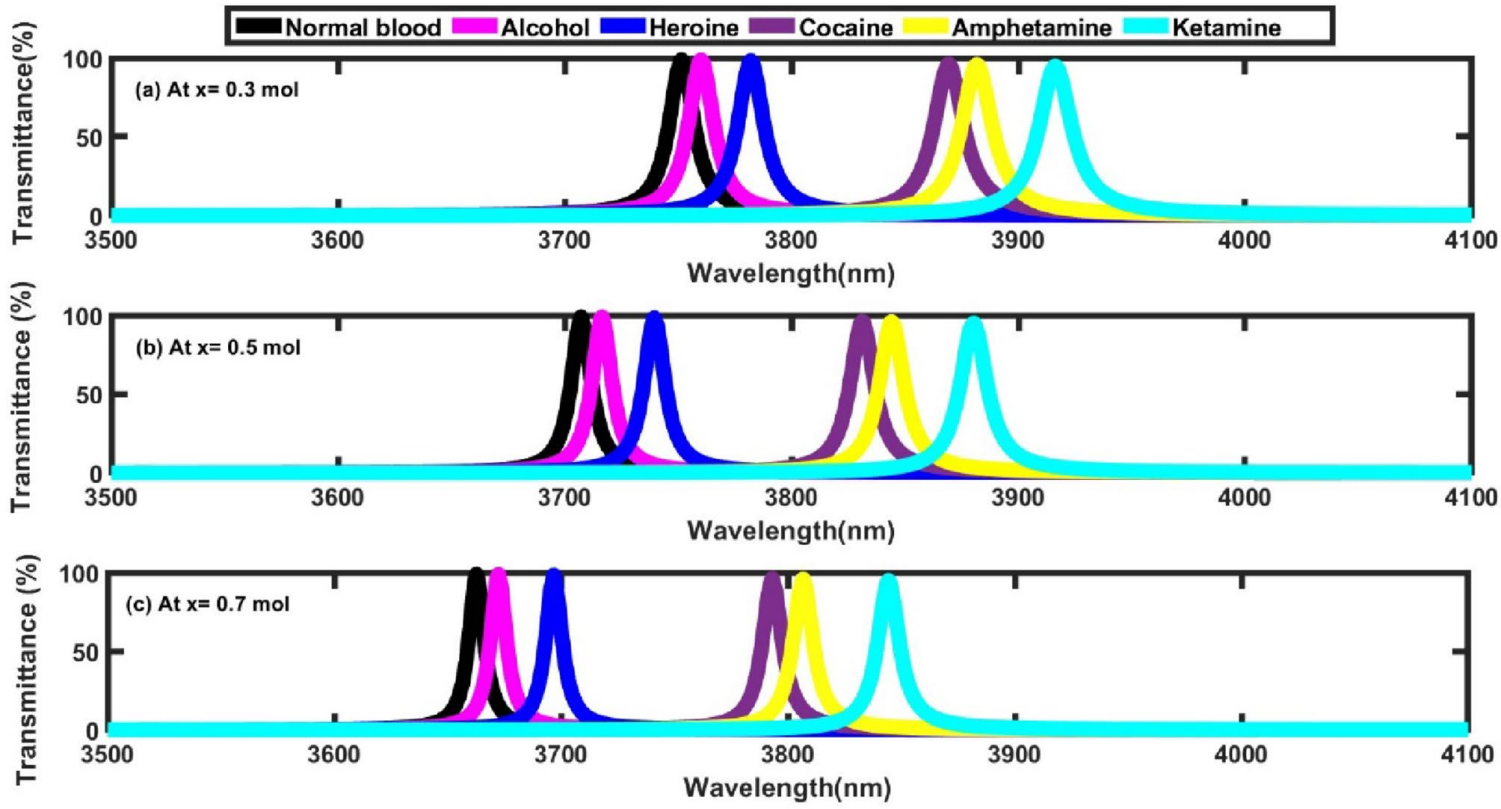
Transmittance spectra of the proposed structure at different values of Al mole fractions, where N=4, and Incident angle = 0°. (**a**) at x= 0.3 mol, (**b**) at x= 0.5 mol, and (**c**) at x=0.7 mol.

**Fig. 5:**
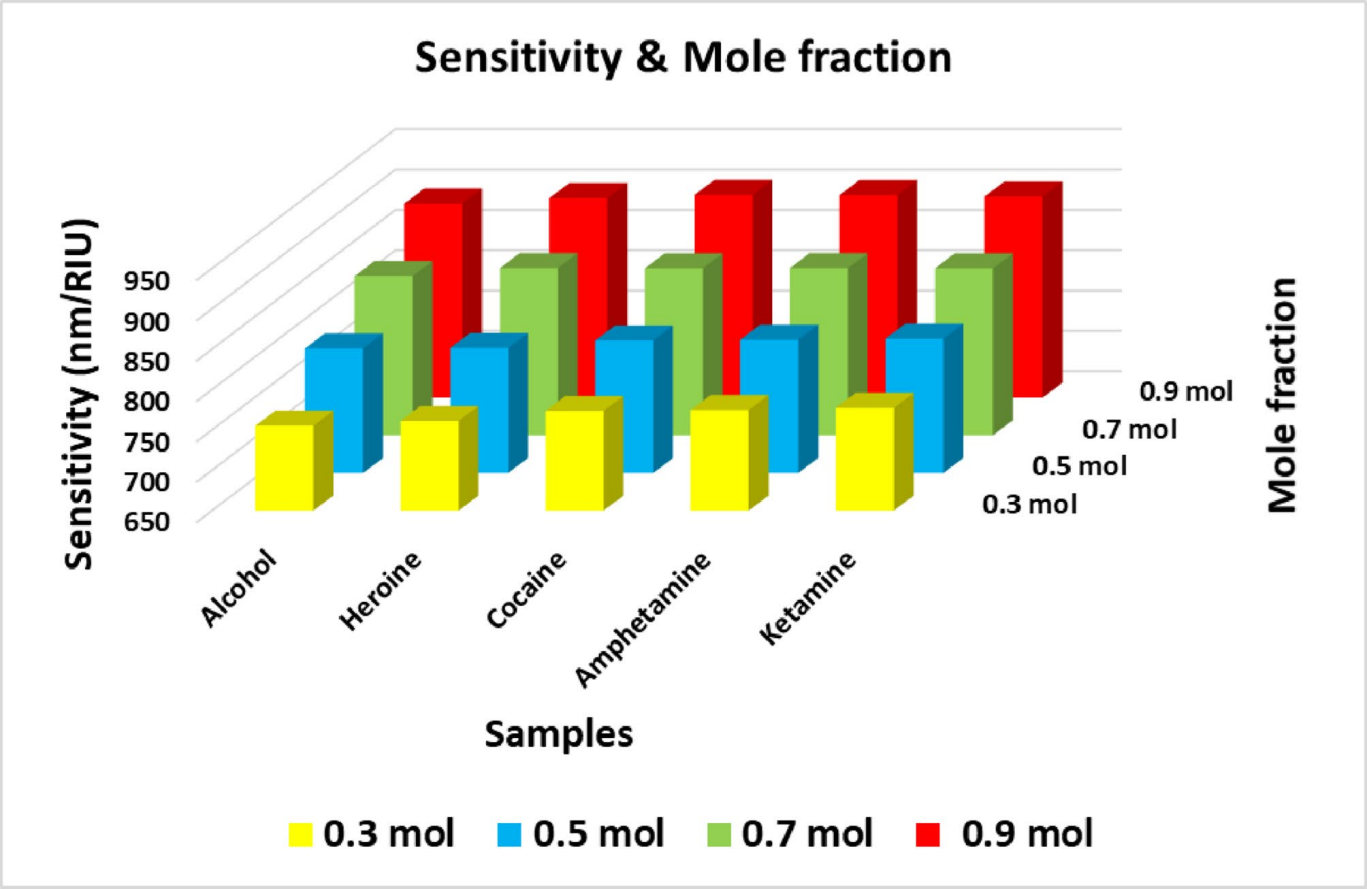
Sensitivity versus Al mole fraction for all illicit drug samples under investigation.

The incorporation of Al_x_Ga_1-x_N quantum dots, a III-Nitride semiconductor, into the photonic crystal sensor structure plays a pivotal role in modulating the sensor’s optical properties. By carefully adjusting the composition ratios of aluminum (Al) and gallium (Ga) through the variation of the aluminum mole fraction (x), the optical properties of these quantum dots can be finely tuned. This incorporation within the biosensor framework enables precise control over optical responses, thereby markedly enhancing essential performance parameters such as sensitivity. Table 3 demonstrates the variation in defect mode position and sensitivity corresponding to different mole fractions of Al (x = 0.3, 0.5, 0.7 mol) within the Al_x_Ga_1-x_N layer when exposed to various illicit drug samples. The results indicate that increasing mole fraction of Al causes a distinct shift in the defect mode position toward shorter wavelengths, as depicted in Fig. 4. Specifically, for normal human blood, the defect mode position decreases from 3751.51 nm at x = 0.3 mol to 3662.67 nm at x = 0.7 mol. A similar pattern is observed when the defect layer is replaced with illicit substances, with the defect mode position consistently shifting as x increases. For instance, in the case of alcohol, the defect mode position shifts from 3760.12 nm (x = 0.3 mol) to 3672.33 nm (x = 0.7 mol). Similar behavior is observed for other substances, including heroin, cocaine, amphetamine, and ketamine, indicating that the increase in Al mole fraction significantly influences the sensor’s optical response. The sensitivity calculated as the change in defect mode position per refractive index unit (nm/RIU), also exhibits a dependency on the mole fraction in Fig. 5. Increasing the mole fraction of Al enhances the sensor’s sensitivity, as evidenced by the rise from 755.26 nm/RIU for alcohol at x = 0.3 to 890.35 nm/RIU at x = 0.9 mol and similarly for the remaining samples. This positive correlation between mole fraction of Al and sensitivity highlights the sensor’s potential for achieving more accurate and dependable detection of illicit substances. Importantly, the mole fraction of 0.9 mol proves to be the optimal value, offering the highest sensitivity among the tested configurations.

**Table 3:** Defect mode positions & sensitivity at different mole fractions of Al.

**Sample**	**At x= 0.3 mol**		**At x= 0.5 mol**		**At x= 0.7 mol**	
	**Defect mode position (nm)**	**Sensitivity (nm/ RIU)**	**Defect mode position (nm)**	**Sensitivity (nm/ RIU)**	**Defect mode position (nm)**	**Sensitivity (nm/ RIU)**
Normal blood	3751.51	_	3707.19	_	3662.67	_
Alcohol	3760.12	755.26	3716.36	804.38	3672.33	847.36
Heroine	3782.03	761.09	3739.46	804.73	3697.04	857.10
Cocaine	3869.25	773.58	3831.1	814.12	3793.09	856.89
Amphetamine	3881.64	774.58	3844.12	815.05	3806.67	857.14
Ketamine	3916.29	777.26	3880.24	816.27	3844.33	856.88

### Effect of incident angle

**Fig. 6:**
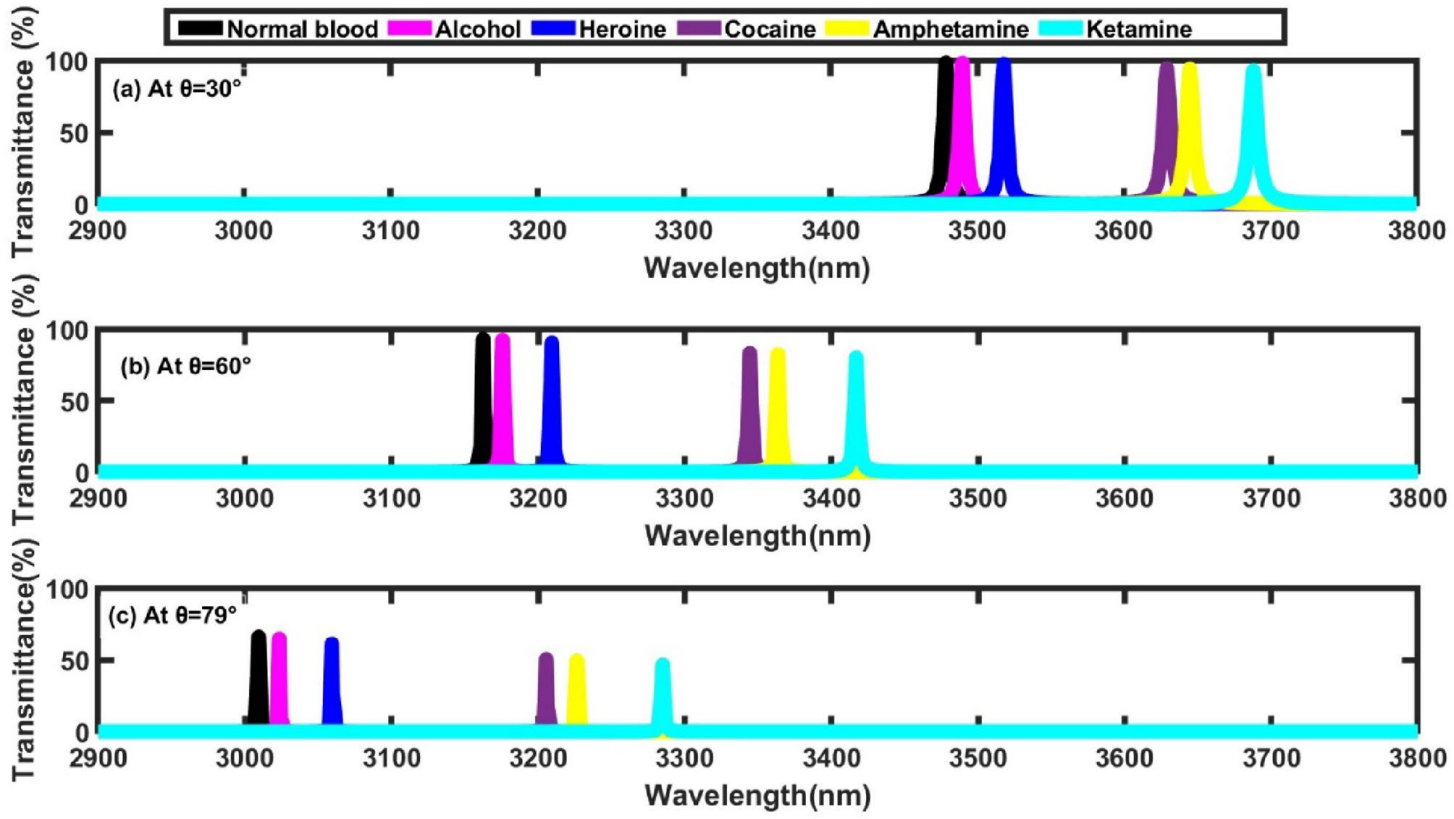
Transmittance spectra of the proposed structure at different values incident angle, where N=4, and x= 0.9 mol. (**a**) at θ_0_=30°, (b) at θ_0_=60°, and (**c**) at θ_0_=79°.

In this subsection, we analyze the effect of varying the incident angle on the performance of the proposed structure, focusing on transmittance intensity, defect mode position, and sensitivity for all samples under investigation. In previous analyses, the incident angle of electromagnetic waves interacting with the designed sensor was fixed at 0°. Here, we expand the investigation by considering incident angles of 30°, 60°, and 79°. The transmittance spectra corresponding to these different incident angles are depicted in Fig. 6. The results clearly indicate that as the incident angle increases, a noticeable blue shift occurs in the defect peaks. This blue shift can be attributed to Bragg scattering theory, which explains the relationship between the incident angle and the Bragg wavelength under transverse electric (TE) polarization as follows^24^:

15 $${\lambda }_{Brg}^{TE}\left(\theta \right)=2\left({\mathrm{d}}_{\mathrm{A}}\sqrt{{\varepsilon }_{{\rm A}}-{\mathrm{sin}}^{2}\theta }+{\mathrm{d}}_{{\rm B}}\sqrt{{\varepsilon }_{\mathrm{B}}-{\mathrm{sin}}^{2}\theta } \right)$$

**Fig. 7:**
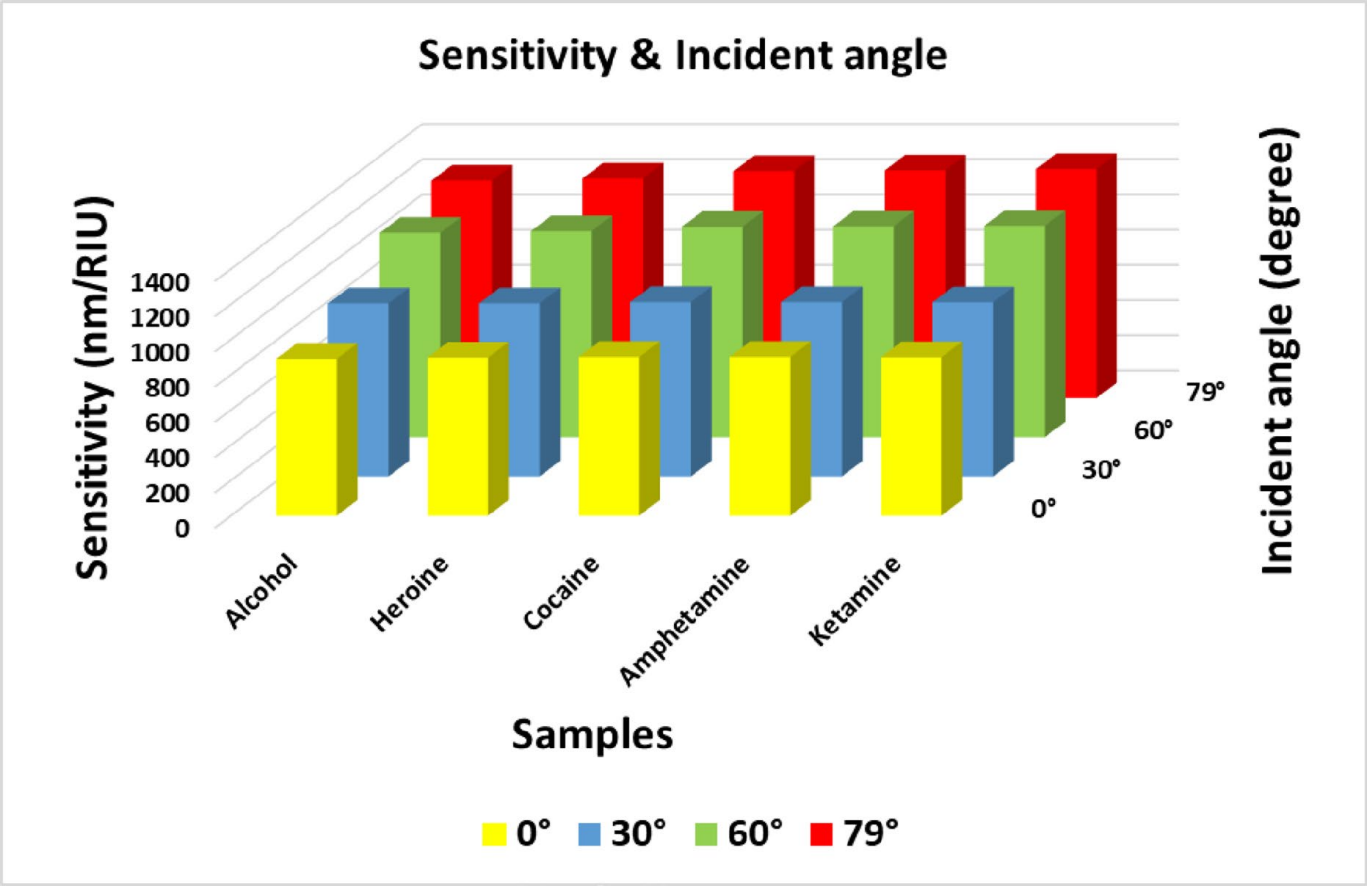
Sensitivity versus incident angles for all illicit drug samples under investigation.

Here ${\lambda }_{Brg}^{TE}\left(\theta \right)$, $\theta$ represent the Bragg wavelength under TE polarization and incident angle respectively. Additionally, from Fig. 6, it is easy to see accurately the relation between transmittance intensity with incident angle change. As the incident angle increases from 30° to 79°, a noticeable decrease in transmittance intensity is observed. This behavior can be explained using Eqs. (4) and (5) presented earlier, which describe the correlation between transmission and the incident angle. Moreover, Table 4 presents the defect mode positions and sensitivities of various samples, including alcohol, heroin, cocaine, amphetamine, and ketamine. Among these substances, ketamine exhibits the highest sensitivity, reaching 1300 nm/RIU at an incident angle of 79°, indicating that the molecular structure and refractive index of each sample significantly affect the sensor’s performance. Furthermore, as depicted in Fig. 7, sensitivity increases markedly with larger incident angles. This observation suggests that adjusting the incident angle can enhance the sensor’s ability to detect subtle changes in refractive index, thereby improving the accuracy of distinguishing between different illicit substances.

**Table 4:** Defect mode positions & sensitivity at different incident angle of light.

**Sample**	**At θ**_**0**_**=30°**		**At θ**_**0**_**=60°**		**At θ**_**0**_**=79°**	
	**Defect mode position (nm)**	**Sensitivity (nm/ RIU)**	**Defect mode position (nm)**	**Sensitivity (nm/ RIU)**	**Defect mode position (nm)**	**Sensitivity (nm/ RIU)**
**Normal blood**	3478.78	-	3162.51	-	3009.42	-
**Alcohol**	3489.98	982.45	3175.74	1160.52	3023.49	1234.21
**Heroine**	3518.19	982.79	3209.41	1169.57	3059.4	1246.38
**Cocaine**	3629.49	990.21	3344.38	1194.94	3205.56	1288.69
**Amphetamine**	3645.1	990	3363.63	1197.14	3226.56	1292.5
**Ketamine**	3688.43	988.91	3416.97	1200.28	3285.02	1300

### Effect of number of periods

**Fig. 8:**
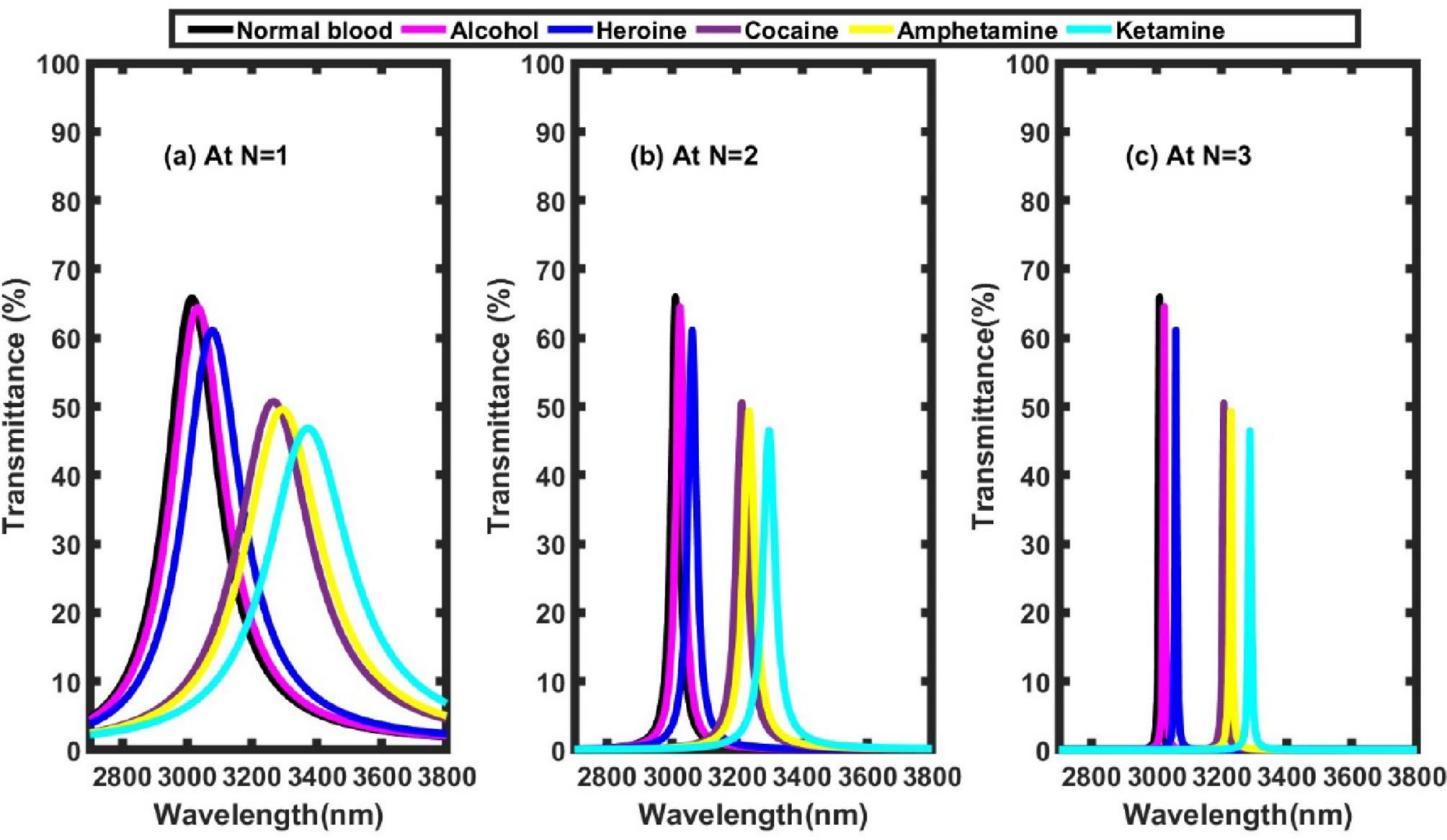
Transmittance spectra of the proposed structure at different values of number of periods, where θ_0_=79°, and x= 0.9 mol. (a) at N=1, (b) at N=2, and (c) at N=3.

**Fig. 9:**
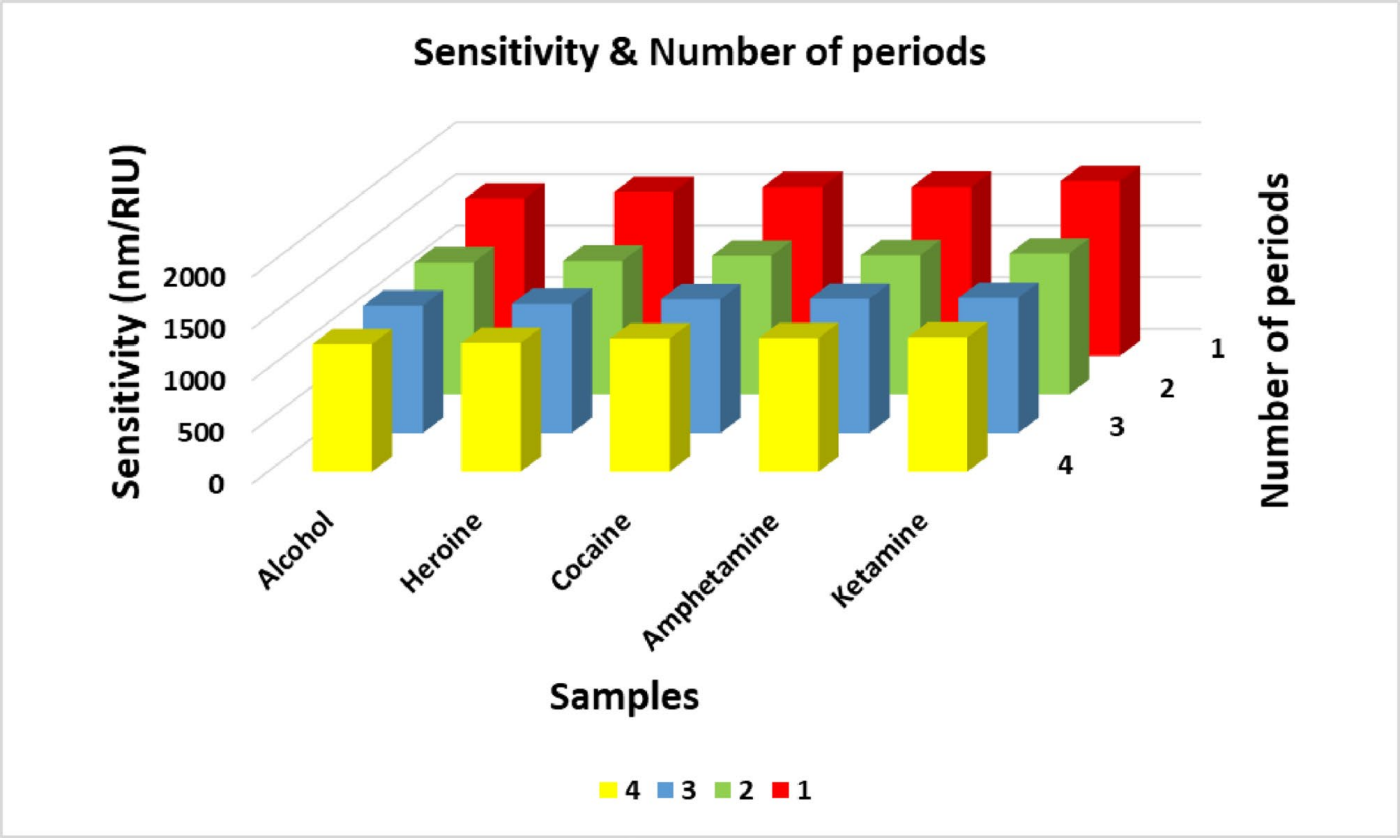
Sensitivity versus number of periods for all illicit drug samples under investigation.

In this subsection, the influence of the number of periods on the characteristics of the defect mode is thoroughly examined. While previous sections maintained a fixed number of four periods, the current analysis explores the transmittance spectra for varying values of N, ranging from 1 to 3, as illustrated in Fig. 8. The corresponding positions of the defect modes and their sensitivities for each case are summarized in Table 5. The results clearly indicate that increasing the number of periods leads to a significant narrowing of the defect mode bandwidth within the transmittance spectrum (Fig. 8). Additionally, the spectral defect mode becomes sharper with higher values of N, as observed in Fig. 8. This behavior can be attributed to the enhanced confinement of electromagnetic waves within the defect layer as the number of surrounding periodic layers increases. Fig. 9 clearly illustrates the influence of the number of periods (N= 1,2,3, and 4) on the sensitivity of the proposed sensor. According to Fig .9 which supported by Table 5’s results, sensitivity exhibits a decline as the number of periods increases. As illustrated in Table 5, ketamine demonstrates the highest sensitivity at N=1 (1690.61 nm/RIU), which decreases to 1308.91 nm/RIU at N=3. This trend is consistent for all investigated substances. Several factors contribute to the observed decline in sensitivity as the number of periods increases. One key factor contributing to this trend is the enhancement of the PBG as more periodic layers are added. This results in stronger light confinement within the defect layer, which in turn reduces the evanescent field’s overlap with the environment. As a consequence, the interaction between the optical field and changes in the external refractive index is weakened. This leads to smaller shifts in the resonance wavelength (Δλ) for a given change in refractive index (Δn), ultimately lowering the sensor’s sensitivity. Secondly, there exists a trade-off between sensitivity and the FWHM of the defect mode. While additional periods contribute to narrower FWHM and sharper resonance peaks due to increased reflectivity, they simultaneously limit the dynamic coupling of the defect mode to external variations. This heightened selectivity of narrow bandwidth comes at the expense of reduced responsiveness to broader ambient refractive index variations. Third, the redistribution of optical energy resulting from increased periodicity leads to enhanced interference and stronger field confinement within the defect layer. This further minimizes the interaction of the confined field with the external environment and external refractive index changes, thereby diminishing the sensor’s sensitivity. Finally, a saturation effect is observed after a certain number of periods, where the optical response of the photonic crystal reaches a saturation point where adding more layers results in a continued improvement of the FWHM and a decrease in the sensor sensitivity^25^ Notably, among all investigated periods, the structure with a single period exhibited the highest sensitivity, identifying it as the optimal design within the framework of this study.

**Table 5:** Defect mode positions & sensitivity at different number of periods.

**Sample**	**At N=1**		**At N=2**		**At N=3**	
	**Defect mode position (nm)**	**Sensitivity (nm/ RIU)**	**Defect mode position (nm)**	**Sensitivity (nm/ RIU)**	**Defect mode position (nm)**	**Sensitivity (nm/ RIU)**
Normal blood	3019.29	-	3010.33	-	3009.63	-
Alcohol	3036.65	1522.80	3024.89	1277.19	3023.7	1234.21
Heroine	3082.85	1585.03	3062.2	1293.51	3059.82	1251.62
Cocaine	3267.94	1633.70	3214.66	1342.50	3206.89	1296.05
Amphetamine	3293.91	1634.64	3236.71	1347.5	3228.1	1300.41
Ketamine	3377.7	1690.61	3299.51	1364.05	3287.12	1308.91

### Effect of defect layer thickness

**Fig. 10:**
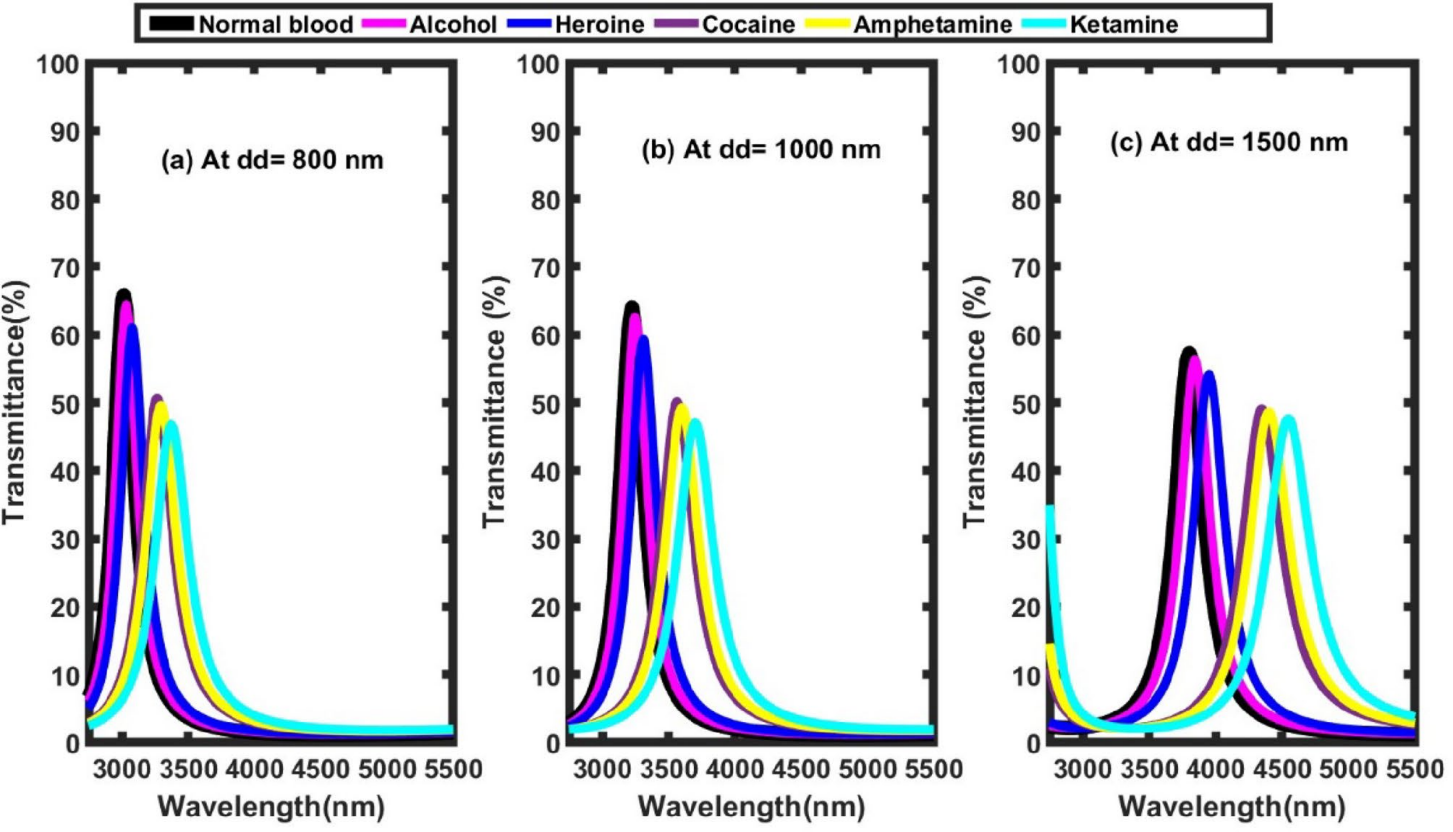
Transmittance spectra of the proposed structure at different values of defect layer thickness, where θ_0_=79°, N= 1, and x= 0.9 mol. (**a**) at dd= 800 nm, (**b**) at dd= 1000 nm, and (**c**) at dd=1500 nm.

**Fig. 11:**
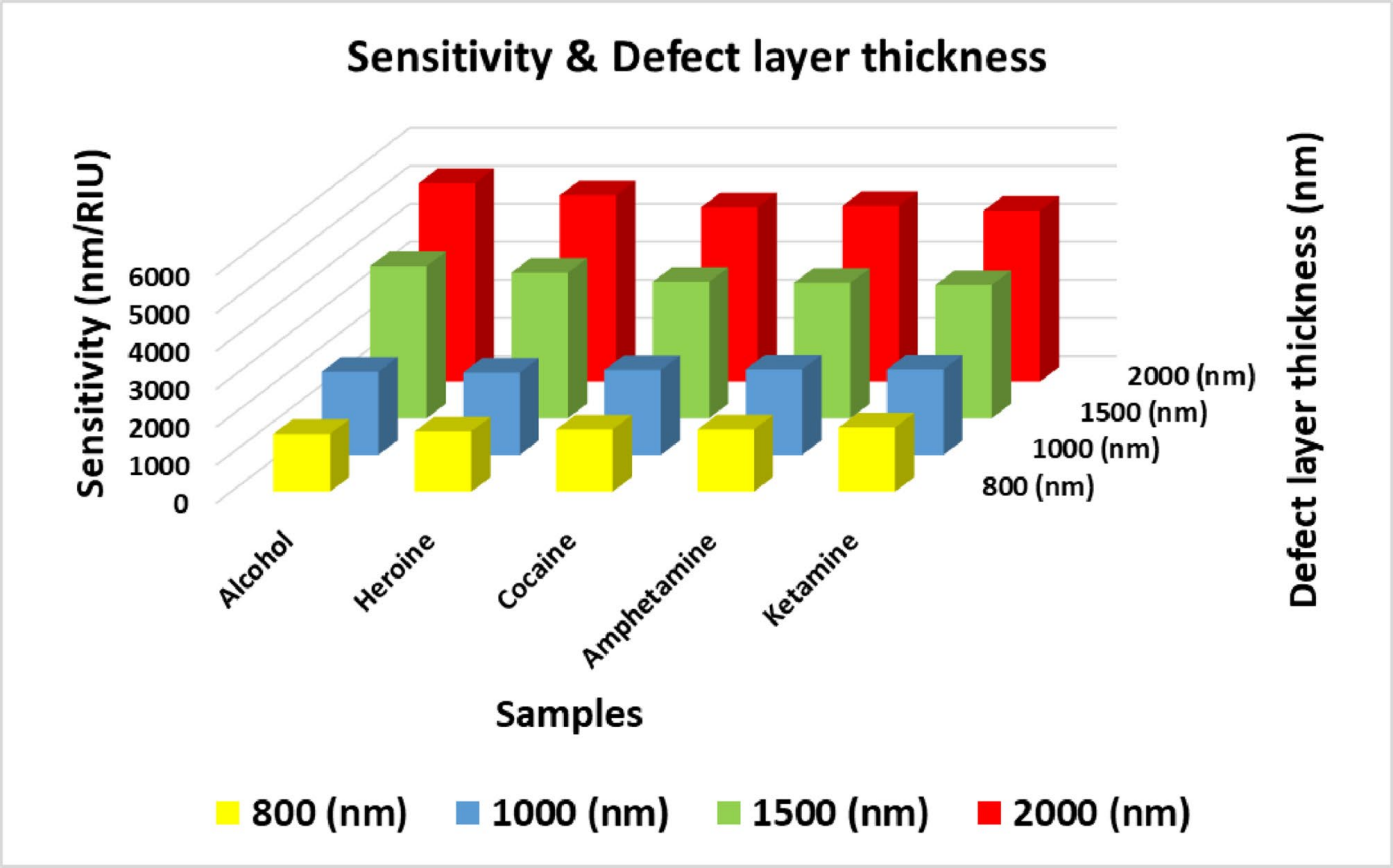
Sensitivity versus defect layer thickness for all illicit drug samples under investigation.

Here, the influence of varying the thickness of the defect layer in the 1D-TPC structure was investigated. This variation was achieved at optimal parameter values of θ₀ = 79°, N= 1, x = 0.9 mol. The effect of the defect layer thickness was examined at values of dd=1000 nm,1500 nm, and 2000 nm, in addition to the baseline defect layer thickness of 800 nm. According to Table 6’s results supported by Fig .10 and Fig. 11, the transmittance spectra in Fig. 10 reveal that increasing the defect layer thickness induces a redshift in the defect mode wavelength, indicating that the resonance condition shifts toward longer wavelengths. This phenomenon is attributed to the increased optical path length within the defect region, which enhances the photon interaction time and alters the resonance conditions. This red shift can be explained according to the previous Eqn. 3. Furthermore, the sensitivity increases with the defect layer thickness, especially at dd=2000nm as shown in Fig. 11, indicating improved sensing capability at higher wavelengths. For instance, the highest recorded sensitivity was for Alcohol at dd=2000nm, reaching 5219.29 nm/RIU, suggesting the structure’s strong potential for accurately distinguishing between various analytes based on their refractive indices.

**Table 6:** Defect mode positions & sensitivity at different defect layer thickness.

**Sample**	**At dd=1000 nm**		**At dd=1500nm**		**At dd= 2000 nm**	
	**Defect mode position (nm)**	**Sensitivity (nm/ RIU)**	**Defect mode position (nm)**	**Sensitivity (nm/ RIU)**	**Defect mode position (nm)**	**Sensitivity (nm/ RIU)**
Normal blood	3222.92	-	3801.14	-	4454.88	-
Alcohol	3247.91	2192.10	3846.78	4003.50	4514.38	5219.29
Heroine	3309.87	2168.32	3954.79	3831.67	4651.44	4901.74
Cocaine	3563.34	2236.66	4347.5	3589.75	5152.44	4583.18
Amphetamine	3601.35	2252.55	4399.65	3562.55	5231.05	4620.05
Ketamine	3700.68	2253.58	4543.78	3503.01	5407.81	4494.95

### Sensor performance parameters

The sensing performance was assessed under the optimized operating parameters of x = 0.9 mol, an incident angle of θ₀ = 79°, a single defect layer (N = 1), and a defect thickness of 2000 nm. As summarized in Table 7, the proposed photonic structure exhibits strong capability for distinguishing among different illicit substances. The analysis reveals that alcohol achieved the highest values of sensitivity, quality factor, figure of merit (FOM), detection resolution (DR), and sensitivity ratio (SR), while simultaneously presenting the lowest signal-to-noise ratio (SNR) and limit of detection (LOD) among all tested samples. In contrast, ketamine showed the weakest performance, characterized by the lowest sensitivity, quality factor, FOM, and DR, along with the highest SNR and LOD values. These outcomes validate the effectiveness and reliability of the proposed photonic crystal sensor for high-precision identification and differentiation of illicit drugs.

**Table 7:** Performance parameters of the proposed structure at optimized values.

**Sample**	**S (nm/ RIU)**	**Q-factor**	**FOM** **(RIU)**^**-1**^	**SNR**	**DR**	**LOD** **(RIU)**	**SR** **(nm)**
Normal blood	-	14.020	-	-	249.923	-	-
Alcohol	5219.29	13.813	15.970	0.182	249.730	0.0031	333.559
Heroine	4901.74	13.288	14.003	0.561	248.620	0.0035	269.644
Cocaine	4583.18	11.543	10.268	1.562	243.890	0.0048	266.173
Amphetamine	4620.05	11.362	10.035	1.686	243.803	0.0049	269.334
Ketamine	4494.95	10.813	8.988	1.905	241.830	0.0055	283.783

### Sensitivity comparison

Finally, the sensitivity achieved by the proposed structure was benchmarked against previously reported photonic sensor designs published between 2017 and 2025. The comparative results, summarized in Table 8, clearly indicate that the proposed sensor exhibits superior performance, achieving a maximum sensitivity of 5219.29 nm/RIU, which highlights its strong potential for ultra-sensitive biophotonic sensing applications. The reported maximum sensitivity corresponds to the optimized physical configuration of the structure, in accordance with established practices in photonic sensor design. Furthermore, the adopted evaluation approach is consistent with recent high-impact studies, including the work cited by the reviewer^26^, where sensor performance is assessed based on resonance wavelength shifts under controlled refractive index variations rather than single-point measurements.

**Table 8:** Comparison study between this work and previous studies.

**Structure used as sensor**	**References**	**Publication Year**	**Sensitivity (nm/ RIU)**
Design and Optimization of Surface Plasmon Resonance-Based Non-Invasive Refractive Index Biosensor Using Ag-SiO_2_ Materials for Alcoholic Beverages Compound Detection.	^27^	2025	1866.66
Rapid Detection of Drugs Abuse by Adapting Surface Plasmonic Micro Ring Resonator.	^28^	2017	3000
TiO_2_ coated tapered optical fiber SPR sensor for alcohol sensing application.	^29^	2023	3250
Current work	-	2025	5219.29

## Limitations and future work

### Limitations

The present study is entirely simulation-based, and all results were obtained using the Transfer Matrix Method (TMM) under ideal optical and material conditions. The sensor performance was evaluated assuming perfectly uniform layers, ideal refractive index values, and noise-free operation. In addition, the sensing analysis was limited to refractive-index variations of drug samples without considering real biological or chemical matrix effects such as temperature fluctuations, surface roughness, fabrication tolerances, material absorption losses, or sample impurities. Moreover, the interaction between the sensing layer and real drug samples was not experimentally validated, and the model does not account for sample preparation constraints or environmental interferences.

### Future work

Future studies will focus on experimental validation using real drug samples to confirm the theoretical predictions presented in this work. Fabrication of the proposed 1D-TPC structure and characterization under laboratory conditions will be essential to evaluate practical feasibility, stability, and repeatability. In addition, future work will investigate material losses, fabrication tolerances, and temperature effects, as well as integration with microfluidic platforms for real-time detection. The performance of the sensor under mixed or contaminated samples will also be explored to enhance its applicability in forensic and biomedical environments.

## Conclusion

In this work, a highly efficient photonic crystal–based sensor was theoretically designed for the detection of illicit drugs using a defective one-dimensional ternary photonic crystal structure. The sensing mechanism is based on tracking the resonance wavelength shifts caused by refractive index variations within the defect layer. The incorporation of PANI, PbS, and AlₓGa₁₋ₓN quantum dots enabled strong light confinement and enhanced interaction between the optical field and the analyte. A comprehensive parametric study was conducted to evaluate the effects of aluminum mole fraction, defect thickness, number of periods, and incident angle on sensor performance. The results revealed that increasing the defect layer thickness and optimizing the incident angle significantly enhance sensitivity, while reducing the number of periods strengthens light–matter interaction. Under optimal conditions, the proposed sensor achieved a high sensitivity of 5219.29 nm/RIU, surpassing many previously reported photonic crystal–based sensors. Furthermore, the device exhibited excellent performance in terms of quality factor, figure of merit, detection limit, and spectral resolution, enabling reliable discrimination between different illicit substances. These findings demonstrate that the proposed structure represents a robust and highly promising platform for future applications in forensic analysis, biomedical diagnostics, and high-performance optical sensing technologies.

## Data Availability

The datasets used and/or analyzed during the current study are available from the corresponding author on reasonable request.
